# Evaluation of Two Novel Hydantoin Derivatives Using Reconstructed Human Skin Model Episkin^TM^: Perspectives for Application as Potential Sunscreen Agents

**DOI:** 10.3390/molecules27061850

**Published:** 2022-03-12

**Authors:** Karolina Słoczyńska, Justyna Popiół, Agnieszka Gunia-Krzyżak, Paulina Koczurkiewicz-Adamczyk, Paweł Żmudzki, Elżbieta Pękala

**Affiliations:** 1 Department of Pharmaceutical Biochemistry, Faculty of Pharmacy, Jagiellonian University Medical College, Medyczna 9, 30-688 Krakow, Poland; justyna.popiol@uj.edu.pl (J.P.); paulina.koczurkiewicz@uj.edu.pl (P.K.-A.); elzbieta.pekala@uj.edu.pl (E.P.); 2 Department of Bioorganic Chemistry, Chair of Organic Chemistry, Faculty of Pharmacy, Jagiellonian University Medical College, Medyczna 9, 30-688 Krakow, Poland; agnieszka.gunia@uj.edu.pl; 3 Department of Medicinal Chemistry, Faculty of Pharmacy, Jagiellonian University Medical College, Medyczna 9, 30-688 Krakow, Poland; pawel.zmudzki@uj.edu.pl

**Keywords:** Episkin, hydantoin derivatives, irritation, permeability, phototoxicity, UV filters

## Abstract

This study aimed to assess two novel 5-arylideneimidazolidine-2,4-dione (hydantoin) derivatives (JH3 and JH10) demonstrating photoprotective activity using the reconstructed human skin model Episkin^TM^. The skin permeability, irritation, and phototoxicity of the compounds was evaluated in vitro. Moreover, the in vitro genotoxicity and human metabolism of both compounds was studied. For skin permeation and irritation experiments, the test compounds were incorporated into a formulation. It was shown that JH3 and JH10 display no skin irritation and no phototoxicity. Both compounds did not markedly enhance the frequency of micronuclei in CHO-K1 cells in the micronucleus assay. Preliminary in vitro studies with liver microsomes demonstrated that hydrolysis appears to constitute their important metabolic pathway. Episkin^TM^ permeability experiments showed that JH3 permeability was lower than or close to currently used UV filters, whereas JH10 had the potential to permeate the skin. Therefore, a restriction of this compound permeability should be obtained by choosing the right vehicle or by optimizing it, which should be addressed in future studies.

## 1. Introduction

Although reasonable ultraviolet (UV) radiation exposure has beneficial effects for human health (e.g., vitamin D synthesis in the skin), overexposure is a main risk factor for the development of both acute effects as well as chronic outcomes [1,2,3]. As a result, photoprotection is one of the most important preventative health approaches, with sunscreen products that contain UV filters as active ingredients representing a key element of photoprotection strategy. However, along with the widespread use of UV filters worldwide, several controversies regarding their usage have appeared [2,4,5,6]. As frequent and repeated application on large skin areas is recommended, even low penetration rates may lead to a significant amount of sunscreen entering the body and appearing in the systemic circulation. This was observed in the case of several frequently used UV filters such as benzophenone-3 (BP3), 3-(4-methylbenzylidene) camphor (4MBC), and octyl-methoxycinnamate (OMC) [7,8,9]. Another limitation of agents allowed for photoprotection is their potential to induce contact and photocontact allergies or phototoxic effects [10,11]. Furthermore, due to their release into the aquatic environment, UV filters and their metabolites are considered an important group of emerging contaminants [12,13]. Therefore, considering the aforementioned facts, there remains a justified need for the development of new, effective UV filters with improved human and environmental safety.

To address this issue, we previously reported the design and synthesis of some novel 5-arylideneimidazolidine-2,4-dione derivatives demonstrating photoprotective activity [14]. The compounds were designed on the basis of the structure of 3-benzylidenecamphor, in which the camphor fragment was replaced with a hydantoin moiety. Other structural modifications involved the introduction of the substituents in the phenyl ring and/or hydantoin fragment as well as extension of the linker by adding an allyl group. These chemical modifications were designed to improve the physico-chemical and photoprotective properties of the tested compounds. The UV absorption parameters of the newly synthesized compounds were comparable with or even more favorable than those of selected commercially available UV filters. Among these compounds diethyl 2,2′-((*Z*)-4-((*E*)-3-(4-methoxyphenyl)allylidene)-2,5-dioxoimidazolidine-1,3-diyl)diacetate (JH3) (Table 1) and diethyl 2,2′-((*Z*)-4-(4-methoxybenzylidene)-2,5-dioxoimidazolidine-1,3-diyl)diacetate (JH10) (Table 1) were the most promising photoprotective agents. These molecules were photostable, exhibited no estrogenic activity in the MCF-7 breast cancer model, no cytotoxicity in selected human skin cells, and were nonmutagenic in the Ames test [14]. Moreover, their metabolic fate with the environmental fungus *Cunninghamella echinulata* was evaluated [15].

As a continuation of our ongoing studies, we have conducted several in vitro tests to assess the new UV filter candidates JH3 and JH10 using the reconstructed human skin model Episkin^TM^. The skin permeability, irritation, and phototoxicity of the compounds were evaluated in vitro. In parallel, their lipophilicity was determined by reversed-phase thin-layer chromatography and their in vitro genotoxicity and metabolism in human liver microsomes model was studied.

## 2. Results

### 2.1. Lipophilicity Assessment

The relative lipophilicity of compounds JH3 and JH10 was determined using reversed-phase thin-layer chromatography. The relative lipophilicity value (*R*_M0_) of JH3 was 3.22 and was greater than that observed for JH10 (*R*_M0_ = 2.46). The calculated log *P* (partition coefficient of octanol-water) values for these compounds were 2.16 for JH3 and 1.41 for JH10 (values calculated by means of the Molinspiration online tool available at https://www.molinspiration.com/cgi-bin/properties; accessed on 15 July 2021).

### 2.2. In Vitro Skin Permeation

#### 2.2.1. In Vitro Skin Permeation Experiment

**Figure 1:**
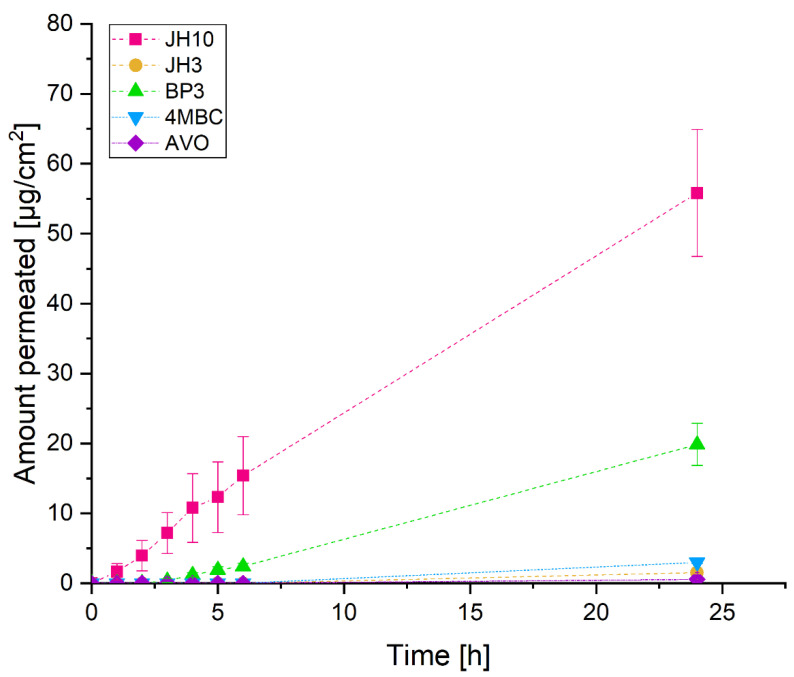
The absorption-time profiles of studied compounds and commercial UV filters incorporated into a formulation (expressed as average percentage ± standard deviation of exposure dose) permeated through Episkin^TM^ following 24 h exposure to 10 mg/cm^2^ (finite dose) of the studied compounds and commercial UV filters: BP3—benzophenone-3, 4MBC—4-methylbenzylidene camphor, AVO—avobenzone. Samples were tested in two independent experiments using three tissues for each chemical.

For the in vitro skin permeation experiments, test compounds and commercial UV filters were incorporated into a formulation. The obtained results showed that the amount of JH10 in the receptor fluid (RF) was detectable (1 µg/cm^2^) after the first hour of exposure. We then observed a constant increase in the portion of JH10 through the next hours. JH3 was undetectable in the RF in the first six hours. After 24 h exposure, the detectable amount of this compound was about 28-fold lower than that of JH10. Among the applied commercial UV filters after 24 h, BP3 demonstrated the highest absorption rate (20 µg/cm^2^), whereas avobenzone (AVO) the lowest (3 µg/cm^2^) absorption rate (Figure 1). The amounts of BP3 in the RF were clearly detectable after the third hour of the epidermis exposure followed by a steady increase over the next 21 h. Similar to JH3, the presence of the commercial UV filter 4MBC was detected only after 24 h exposure (Figure 1).

**Figure 2:**
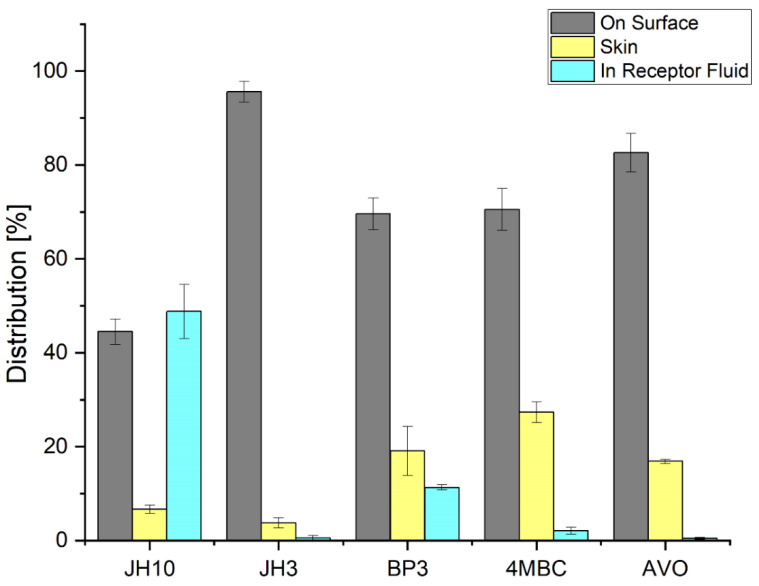
Distribution of test compounds and commercial UV filters incorporated into a formulation (expressed as average percentage ± standard deviation of exposure dose) following 24 h exposure to 10 mg/cm^2^ (finite dose) of the studied compounds and commercial UV filters: BP3—benzophenone-3, 4MBC—4-methylbenzylidene camphor, AVO—avobenzone. Samples were tested in two independent experiments using three tissues for each chemical.

Figure 2 shows the distribution of test compounds and commercial UV filters after permeation through the Episkin^TM^ following 24 h exposure to 10 mg/cm^2^ (finite dose) of the studied compounds. According to the results obtained, JH10 showed the highest cumulative absorption with 48% of the applied dose detected in the RF. By contrast, the absorbed amount of JH3 was 1%. In the case of the studied commercial UV filters, absorbed fractions of 12% and 3% were observed for BP3 and 4MBC, respectively. The absorbed amount of AVO was very similar to JH3 (i.e., about 1%). The epidermis analysis resulted in recovery of 28, 18, 16, 7, and 3% of the applied dose of 4MBC, BP3, AVO, JH10, and JH3, respectively. The amount of test compounds remaining on the surface of the Episkin^TM^ reached 95% for JH3 and 45% for JH10. For the commercial UV filters, the values were in the range of 70–83% and were the highest for AVO. The amount of BP3 in this compartment was comparable with 4MBC.

#### 2.2.2. Method Validation

The developed UPLC/UV-Vis method was specific to the examined compounds and guaranteed obtaining well-shaped peaks. The peaks were well-resolved on all the chromatograms (R > 2). MS analysis confirmed the identity and purity of the obtained peaks.

Based on regression analysis and Mandel’s fitting tests (*p* > 0.1), it was assumed that the calibration data fit well with the linear models for all the investigated compounds. The correlation coefficient and determination coefficient (r^2^) obtained for the models were over 0.999. The distribution of the residuals could be approximated with a normal distribution as it is shown by *p* value of the normality test (Shapiro–Wilk)—*p* > 0.2.

The sensitivity of the method was good. The limit of detection (LOD) and limit of quantification (LOQ) values for the compounds were found to be below 0.10 and 0.30 μg/mL, respectively. Good precision and intermediate precision with %RSD less than 6% were observed. The ANOVA test showed no significant differences between analyses in different days (*p* > 0.3). The accuracy of the obtained method was satisfying and was in a range of 98.9–103.2% for all the compounds. Recovery for the analyzed compounds was good and was in the range of 90.7–98.7%. In all the deliberately varied chromatographic conditions (flow rate, column temperature, mobile phase composition), the examined compounds were adequately resolved, and the order of elution remained unchanged. Regression analysis results are presented in Table 2 and Table 3.

### 2.3. In Vitro Skin Irritation

**Figure 3:**
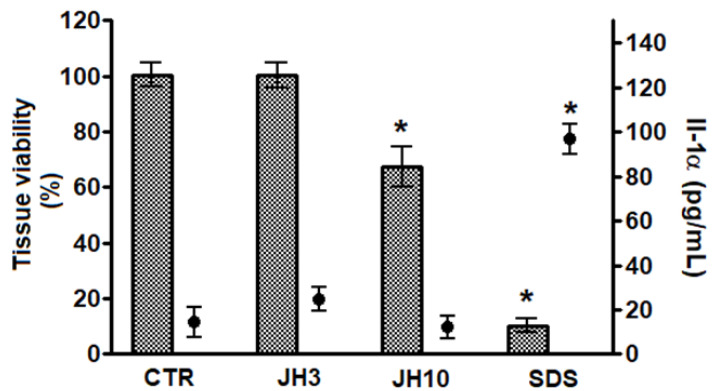
Compounds JH3 and JH10 in vitro evaluation of skin irritation using Episkin^TM^ model. Results are expressed as mean ± standard deviation, samples were tested in two independent experiments using three tissues for each chemical, positive, or negative control. (*) *p* < 0.05 when compared to negative control. 5 µL of sterile water was applied onto the surface of the epidermis, followed by the application of 26 mg/cm^2^ of the formulations of JH3 and JH10; sodium dodecyl sulfate (SDS) (5% in water) and PBS were used as positive and negative (CTR) controls, respectively. Both controls were applied topically (26 mg/cm^2^).

After topical application of formulations containing test compounds JH3 or JH10 on the Episkin^TM^ system, the reduction of mean tissue viability below 50% was not observed (Figure 3), therefore the test compounds can be classified as non-irritants. According to the OECD prediction model and Episkin protocols, there is a 50% cut-off value of cell viability [16,17]. Moreover, no significant increase with the second irritation marker, i.e., Il-1α, was observed (Figure 3). Sodium dodecyl sulphate (SDS), a positive control chemical, resulted in a significant cell viability decrease reaching approximately 10% as compared to negative control samples, as well as significant Il-1α release (i.e., 96 pg/mL).

### 2.4. In Vitro Skin Phototoxicity

**Figure 4:**
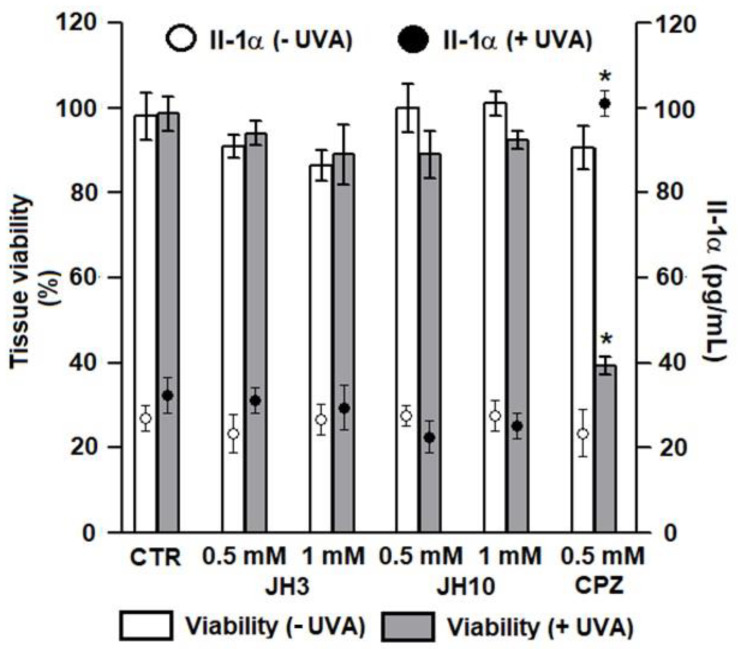
In vitro phototoxic potential of compounds JH3 and JH10 on Episkin^TM^ model. Results are expressed as mean ± standard deviation, samples were tested in two independent experiments using three tissues for each chemical, positive, or negative control. (*) *p* < 0.05 when compared to non-irradiated epidermis. JH3 and JH10 (final concentrations of 0.5 and 1 mM) solubilized in ethanol (final concentration of 1% in water) were applied topically on the tissue surface; chlorpromazine (CPZ, positive control) final concentration was 0.5 mM, solvent alone served as a negative control (CTR).

The study demonstrated that without UVA exposure, no changes in tissue viability were noticed after test compounds JH3 and JH10 were applied onto Episkin^TM^ with the two concentrations (i.e., 0.5 and 1 mM) tested. There was also no significant difference in the viability of irradiated epidermis treated with the same test concentrations (Figure 4). Measurement of the Il-1α confirmed these results, as its level reached a maximal value of 31 pg/mL and corresponded to the release observed in non-irradiated tissue (Figure 4). These results demonstrated the absence of phototoxic potential for test compounds in our test conditions. Chlorpromazine (CPZ), which served as a positive control, in the tested concentration of 0.5 mM after UVA exposure significantly decreased tissue viability to about 40% as compared to non-irradiated epidermis. Moreover, the Il-1α release was correlated with cell mortality after CPZ treatment and UVA exposure (Figure 4).

### 2.5. Cytotoxicity to CHO-K1 Cells

**Figure 5:**
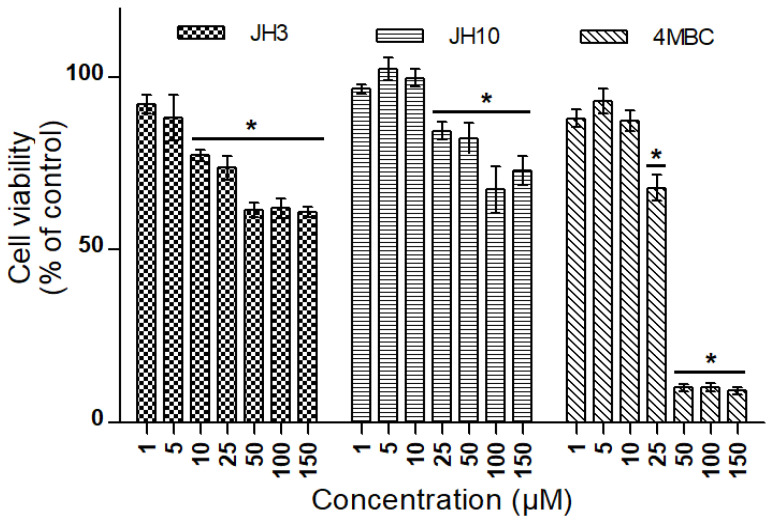
Cell viability in CHO-K1 cells exposed to JH3, JH10, and 4-methylbenzylidene camphor (4MBC) determined by MTT assay. Graph represents percentage of viable cells in comparison to the control condition ± standard deviation (* *p* < 0.05). Experiment was conducted two times with three repetitions for each condition.

In the MTT test, a reduction in CHO-K1 cell viability was observed following a 24 h treatment at doses of 10 to 150 µM of JH3, 25 to 150 µM of JH10 and 4MBC in comparison with the negative control group (Figure 5).

### 2.6. Micronucleus Assay

In the normal and vehicle control groups, the mean values of micronuclei (MNs), dicentric bridges (DBs), nuclear buds (NBs), and nuclear division index (NDI) were 8.2 ± 3.1 and 9.3 ± 4.3; 5.5 and 4.6; 4.1 and 5.2; 1.45 and 1.41, respectively (Table 4). Treatment of CHO-K1 cells with increasing concentrations (1, 5 and 10 µM) of JH3, JH10, and 4MBC did not significantly alter any of the evaluated parameters. Mitomycin C (MMC, 0.5 µg/mL), a positive control for this assay, promoted an approximately 10-fold increase in the number of MNs (*p* < 0.05), a 5-fold increase in the number of DBs (*p* < 0.05), and a 7-fold increase in the number of NBs (*p* < 0.05) when compared to the normal and vehicle control groups. The NDI for MMC was of 1.11 (Table 4).

### 2.7. In Vitro Microsomal Biotransformation in Human Liver Microsomes

**Figure 6:**
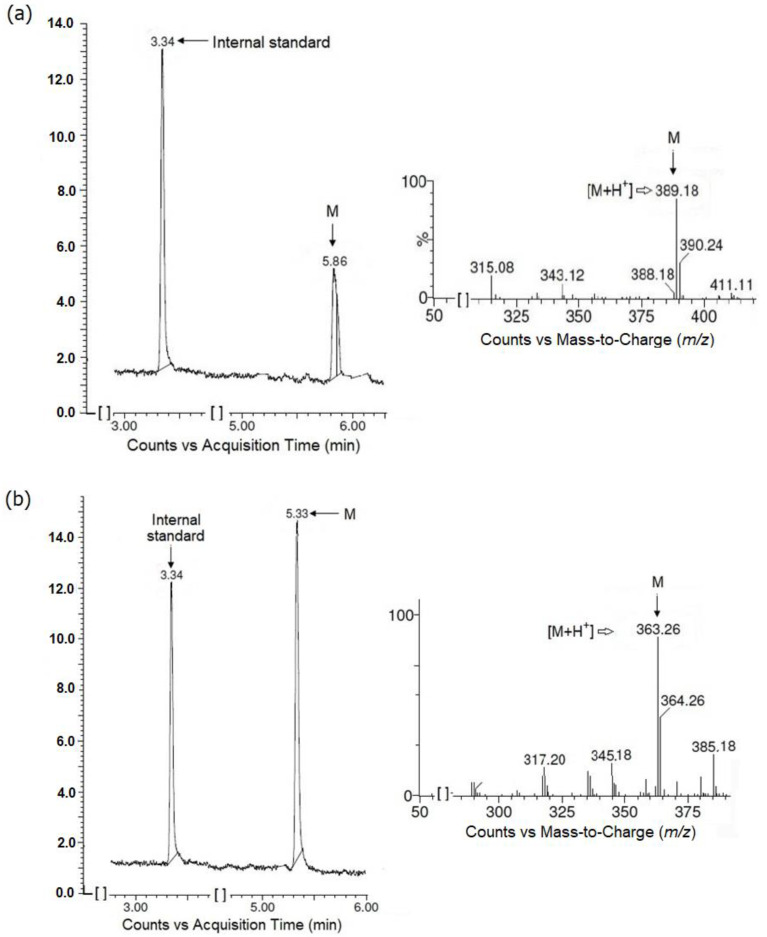
Formation of main metabolites (M) from JH3 (**a**) and JH10 (**b**) when test compounds were incubated with human liver microsomes (HLMs) without NADPH-regenerating system. Chromatograms and ion fragmentation (MS/MS) spectra for main metabolites are shown.

Initially the tested esters of hydantoin(di)acetic acid derivatives (JH3 and JH10) were incubated in a buffer (pH = 7.4) in the absence of human liver microsomes (HLMs) and demonstrated no signs of spontaneous hydrolysis after 30 min incubation at 37 °C. Secondly, the studied compounds were incubated with HLMs in the absence or presence of NADPH. In both situations the test compounds were biotransformed to one major metabolite (M, parent compound losing 28 Da), coming from the cleavage of the ester bond. The JH3 metabolite was eluted at 5.86 min, with a monoisotopic mass of *m*/*z* 389 ([M+H]^+^), whereas the JH10 metabolite retention time was 5.33 min, with a precursor mass of *m*/*z* 363 ([M+H]^+^) (Figure 6).

**Figure 7:**
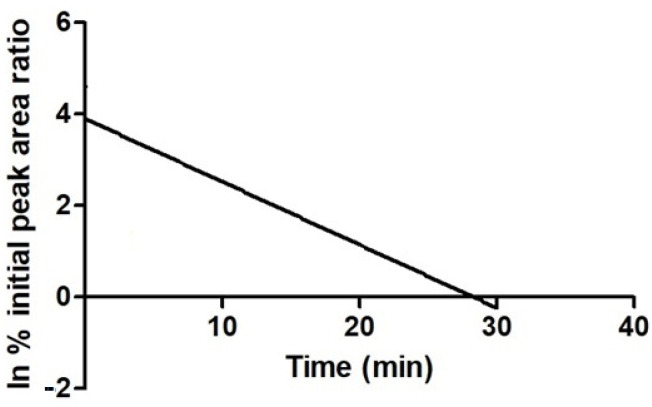
Plot demonstrating the depletion of JH3 in human liver microsomal system. Parent depletion expressed as natural logarithm of the percent remaining as peak area ratio (parent/internal standard).

Approximately 5% of the parent compound JH3 remained at the 15 min time point, resulting in an in vitro half time (t_1/2_) of 5 min and intrinsic clearance (Cl_int_) of 172 µL/mg min. A plot demonstrating the depletion of JH3 in HLMs is given in Figure 7. For JH10, less than 0.5% of this compound was detected at the 5 min time point. Due to the limited number of time points in this region, the t_1/2_ of JH10 could not be calculated accurately, however, it can be stated that its t_1/2_ was lower than 5 min and its Cl_int_ was higher than that of JH3 (Table 5).

## 3. Discussion

When analyzing cosmetic ingredients exposure, percutaneous absorption data are highly desired for safety reasons. To ensure effectiveness, UV filters should stay on the skin surface to enable absorption of UV radiation. Moreover, these preparations should not penetrate into the viable epidermis, the dermis, and into the systemic circulation.

The present study demonstrated the percutaneous absorption data of the new UV filter candidates JH3 and JH10 and three reference UV filters (BP3, 4MBC, and AVO) using the in vitro Episkin^TM^ model. In order to mimic human exposure, test substances were incorporated into a cosmetic preparation. Therefore, we manufactured a base formulation containing test substances in the concentration of 2%.

According to OECD guidelines, finite dose conditions of exposure should be used during in vitro percutaneous absorption studies, as under normal conditions of human exposure to chemicals, finite doses are usually encountered [18,19]. However, a wide range of application doses with different exposure times can be seen in the literature [20,21,22,23,24,25]. Under finite dose conditions, data defining the quantity of a chemical compound present in the receptor fluid, the amount associated with the skin, and the quantity washed from the skin can be accurately determined [19]. Within the present study, the finite dosing scenario was employed [26,27] with a compound dose of 10 mg/cm^2^ and 24 h exposure conditions.

Although the skin consists of three layers, the epidermis, dermis, and the hypodermis, the uppermost layer of epidermis, *stratum corneum*, represents the main barrier for percutaneous absorption of substances. Commercially available, reconstructed human epidermis (RHE) models, including Episkin^TM^, are similar in their morphology, lipid composition, and biochemical markers to native human tissue [28]. These models have been indicated as an alternative to human skin samples and a relevant model for in vitro permeability experiments [20,21,23,28,29,30,31,32]. However, one of the limitations of RHE models is their weaker barrier function compared to natural human skin [20,23,31,32,33]. Despite this drawback, previous papers confirmed that the data regarding permeability rank order of compounds are reliable [28,29].

Parameters such as lipophilicity and molecular weight are key factors affecting the dermal penetration of chemical compounds. Skin permeation requires balanced aqueous and lipid solubility. According to the literature, to pass through the skin a compound should possess the log *P* (partition coefficient of octanol-water) value in the range of 1–3, and its molecular weight should be lower than 500 [34,35,36]. The present study indicated a higher epidermis permeability of JH10 compared to JH3 and the three reference UV filters. In the investigation the decreasing permeation pattern was in the following order: JH10, BP3, 4MBC, and JH3 and AVO with comparable values. This rank order could be partly explained by the physico-chemical properties of the tested compounds. In the case of JH10, its mass is 390 g/mol, that is lower than 500, and its experimentally determined relative lipophilicity parameter (*R*_M0_) is 2.46 (calculated log *P* = 1.41). As regards JH3, its *R*_M0_ is greater than 3 (*R*_M0_ = 3.22, calculated log *P* = 2.16), and its mass (i.e., 416 g/mol) is slightly higher than that of JH10. The commercial UV filters, despite possessing a relative low mass (228, 254, and 310 g/mol for BP3, 4MBC and AVO, respectively) were characterized with higher log *P* values (3.79, 4.95, and 4.51 for BP3, 4MBC, and AVO, respectively [37,38], and thus higher lipophilicity. BP3 exhibited the highest and AVO the lowest capacity to permeate the RHE. This was generally in line with previous results which demonstrated that BP3 itself had a good ability to permeate and penetrate the skin; whereas the permeated amounts of AVO were below the lowest limit of quantification [24,25,39,40]. Based on the obtained experimental data, compound JH3 would be more adequate as a UV filter because of its lower permeability.

More lipophilic compounds tend to accumulate in the lipid phases of the *stratum corneum* and have difficulty in penetrating the viable hydrophilic epidermis. This is consistent with the compound distribution analysis, as 4MBC was characterized with the highest barrier (Episkin^TM^) recovery. The amounts of JH3 and JH10 in the epidermis were lower than those of the commercial UV filters. The present study showed that the more hydrophilic JH10 demonstrated higher absorbed fractions than other more lipophilic compounds, which tend to accumulate within the lipid-rich *stratum corneum*. This is consistent with previous observations by Abdallah et al. [23] and Abou-Elwafa Abdallah et al. [32] who investigated human dermal absorption of some polybrominated diphenyl ethers and chlorinated organophosphate flame retardants using the Episkin^TM^ model.

UV filters are designed for external application on the outermost layers of the skin. An ideal sunscreen should exhibit a high accumulation in the *stratum corneum*, but minimal permeation into the deeper parts of the skin to avoid its systemic exposure with unknown consequences [41,42]. Besides the compound’s physico-chemical parameters such as lipophilicity and molecular weight, the nature of the vehicle by which the sunscreen is applied and the time of exposure are also important when analyzing skin permeability [25,41,43,44,45,46,47]. As JH10 was characterized with relatively high dermal bioavailability when compared with the other compounds under study, its cutaneous penetration should be modified in future studies by designing its optimal formulation. It was demonstrated previously that such modifications can significantly influence a compound’s skin permeation [41,43,44,45,46,47]. It should also be taken into consideration that the penetration of molecules is higher in the RHE than in viable human skin [48].

When new active cosmetic ingredients are developed, the skin irritation potential is evaluated to identify substances capable of inducing adverse skin reactions [49]. It is noteworthy that acute skin irritation is the most common local toxic effect observed after contact with cosmetic ingredients and is characterized by reversible damage of the skin [50]. In the present study, two parameters were determined in the assay based on the Episkin^TM^ model, such as cell viability and the amount of cytokine Il-1α released into the culture media. The obtained results identified that both JH3 and JH10 can be classified as non-irritants.

Cell viability and the amount of Il-1α release were also assessed to determine JH3 and JH10 phototoxicity (photoirritation). It is a skin inflammatory reaction that occurs after a single contact with a photoreactive chemical and subsequent exposure to light, especially UVA radiation [51]. Phototoxicity testing is a key element in evaluating the safety of new cosmetic ingredients. The results confirmed the lack of phototoxic potential of test compounds JH3 and JH10.

Genotoxic compounds can damage the genetic material of the cell directly or indirectly by interacting with non-DNA targets leading to genotoxic effects [52,53]. Varying types of DNA damage can be observed, ranging from gene to structural or numerical chromosome changes. Due to the fact that routinely used biological and chemical agents such as UV filters impact human health, the data on their genotoxicity has become an important issue. The micronucleus assay constitutes a valuable tool for the in vitro testing of the genotoxicity of substances. The test is based on the counting of micronuclei (MN), that is a small, extranuclear body formed in dividing cells from acentric chromosome/chromatid fragments or whole chromosomes/chromatids during the metaphase/anaphase transition of mitosis. Bridges linking nuclei on the binucleated cells (DB) and buds in the main nucleus (NB) that provide an additional evaluation of the chromosomal rearrangements are also scored [54,55,56].

Initially, the cytotoxicity of JH3, JH10, and a reference UV filter 4MBC in CHO-K1 cells was evaluated to define the range of concentrations of the tested compounds to be used in the assessment of their genotoxicity. In the MTT test, a 24 h treatment with JH3 reduced CHO-K1 cells’ viability at doses 10 µM and higher, whereas both JH10 and 4MBC produced a significant reduction of cell viability at doses higher than 10 µM. Due to the fact that in all the compounds screened in the MTT test the viability of cells at concentrations ranging from 1 to 10 µM still exceeded 75%, these concentrations were applied in the genotoxicity evaluation.

The present study documented no changes in the number of MN, DB, and NB in CHO-K1 cells treated with the target compounds and 4MBC. Additionally, treatment with these substances did not interfere with cell proliferation, as the nuclear division index (NDI) of the treated cells remained unchanged compared to the control. The fact that the test compounds did not cause DNA damage in the MN assay corroborates the mutagenicity results found formerly in the Ames test. It was observed that JH3 and JH10 are not base substitution or frameshift mutagens [14]. Moreover, our results were consistent with some previous studies that showed negative effects of 4MBC in both the bacterial mutation (Ames) assay and the in vitro chromosomal aberration test [57].

UV filters are designed for external application on the skin; however, previous studies have shown that they can be absorbed through the skin, further metabolized, and eventually bioaccumulated or excreted [58]. To more thoroughly assess the effects of the human exposure to such compounds, it is important to study the pathways by which they are biotransformed in the body [40,59]. HLMs represent an affordable in vitro alternative for xenobiotics such as drugs and cosmetic ingredients biotransformation studies. Liver microsomes are a rich source of enzymes responsible for the metabolism of the majority of drugs through phase I allowing for the identification of metabolites before their further transformation in phase II reactions.

The current study was designed to preliminarily examine the metabolism of target compounds in humans in vitro. It was demonstrated that JH3 and JH10 hydrolysis appears to constitute their important metabolic pathway. Both compounds were predominantly transformed to ester hydrolysis products in HLMs, both in the presence or absence of an NADPH-generating system. Therefore, it may be concluded that enzymes other than the cytochrome P450 (CYP450) superfamily have a great contribution to their metabolism. These findings were in agreement with our previous studies of the metabolism of target compounds with rat liver microsomes (RLMs) [14]. Lee et al. [60] confirmed that, in terms of different metabolic enzyme activities, RLMs often closely resemble HLMs. However, additional and more advanced studies (e.g., using specific enzyme inhibitors) are needed to further explore the hydrolysis and to investigate more thoroughly the test compounds’ metabolism.

Ester-containing drugs are most frequently cleaved by carboxylic ester hydrolases (E.C.3.1.1) with a prominent role of carboxylesterases (CES, E.C.3.1.1.1) compared with the other esterase subfamilies or CYP450 [61,62,63]. It is well established that the liver microsomal fraction contains the highest activity of esterases, but there are large species differences, especially among small laboratory animals [64]. The present study showed that both JH3 and JH10 were degraded in HLMs, resulting in very short in vitro microsomal half-lives (*t*_1/2_∼5 min) and subsequently high in vitro Cl_int_ (>172 µL/mg min).

## 4. Materials and Methods

### 4.1. Test Compounds

The compounds denoted as JH3 and JH10 were synthesized in the Department of Pharmaceutical Biochemistry and Department of Bioorganic Chemistry, Chair of Organic Chemistry, Faculty of Pharmacy, Jagiellonian University Medical College. The chemical structures and purity of these compounds were established on the basis of spectral data (^1^H-NMR and LC/MS analyses; JH3: C_21_H_24_N_2_O_7_, purity: 100%, CAS number: 2377854-57-2; JH10: C_19_H_22_N_2_O_7_, purity: 100%, CAS number: 2377854-49-2). The chemical synthesis and physico-chemical data of both compounds were discussed in a separate paper [14].

For the in vitro skin permeation and irritation experiments, test compounds as well as commercial UV filters (used as reference standards) were incorporated into a formulation with a concentration of 2%. The final composition of the formulation was as follows: Tween 20 43.2%, water 37.5%, triacetin 4.8%, liquid paraffin 2.8%, stearic acid 2.3%, test compound/reference UV filter 2.0%, cetyl alcohol 1.7%, Cetiol A 1.1%, isopropyl myristate 1.1%, propylene glycol 0.9%, Speziol C16-18 0.9%, Tween 60 0.9%, triethanolamine 0.7%, and Carbopol 934 0.1%. The Tween 20, Tween 60, triacetin, stearic acid, cetyl alcohol, and isopropyl myristate were purchased from Sigma Aldrich (Darmstadt, Germany). The liquid paraffin, propylene glycol, and triethanolamine were purchased from Chempur (Piekary Śląskie, Poland). The Cetiol A and Speziol C16-18 were obtained from BASF (Ludwigshafen, Germany), whereas the Carbopol 934 was provided by Lubrizol (Wickliffe, OH, USA).

### 4.2. Lipophilicity Estimation

The experimental log *P* was determined by reversed-phase thin-layer chromatography (RP-TLC). TLC was carried out on plates precoated with silica gel RP-18 F_254_ (Merck, Darmstadt, Germany). The mobile phases used were the mixtures of methanol (commercially available material of reagent grade) and 100 mM potassium–phosphate buffer (pH = 7.4), with the methanol content ranging from 95 to 65% (*v*/*v*) in 5% increments. A total of 10 μL of test compound solutions in methanol (1 mg/mL) were spotted on the plates. The plates were developed in chromatographic chambers previously saturated with solvent for 1 h. After development (at room temperature in a normal chamber: 22 × 22 × 7 cm) and the drying of the plates, the spots were observed under a UV lamp (λ = 254 nm) [65]. The *R*_f_ values were mean values from two independent chromatographic runs. The experimental *R*_f_ was converted into the *R*_M_ value according to the following equation [66]: *R*_M_ = log (1/*R*_f_ − 1). The calculated values were extrapolated for a 0% methanol concentration (*R*_M0_) using the equation: *R*_M_ = *R*_M0_ + *bC*, where *C* is the concentration (%, *v*/*v*) of methanol in the mobile phase and *b* is the regression term.

### 4.3. Episkin^TM^ Reconstructed Human Epidermis

The Episkin^TM^ model (aged 13 days, large/1.07 cm^2^ surface area) was provided by Episkin (Lyon, France). The Episkin^TM^ kit consists of 12 reconstructed epidermis units made of type I collagen matrix, representing the dermis, surfaced with a film of type IV collagen, upon which is laid a stratified and differentiated epidermis derived from human keratinocytes [17]. Upon receipt of the tissues, the culture inserts were removed from the agarose-nutrient solution and transferred into 12-well plates containing 2 mL of the maintenance medium (MM) per well. The MM was provided by the manufacturer. Tissues were then incubated for 24 h at 37 °C in a humidified atmosphere of 5% CO_2_ before use in the permeation, irritation, or phototoxicity experiments.

#### 4.3.1. The Episkin^TM^ Skin Permeation Test Method

##### Skin Permeation

Prior to the experiments, the Episkin^TM^ inserts (aged 13 days, large/1.07 cm^2^ surface area) were washed with sterile phosphate buffered saline (PBS; Lonza, Basel, Switzerland) to remove all traces of the MM and placed in new 12-well plates containing 2 mL of PBS + Tween 20 (0.25% *w*/*w*) (Sigma-Aldrich, Darmstadt, Germany) as the receptor fluid. The receptor fluid composition was based on the pilot experiments. Samples were tested in two independent experiments using three tissues for each chemical.

The formulations of the test compounds (JH3 and JH10) and commercial UV filters were applied onto the skin surface in the donor compartment in a net dose of 10 mg/cm^2^ (finite dose application) using disposable flexible loops. To quantify the dose of the formulation applied on the top of the Episkin^TM^ model, the loops were weighed, filled with approximately 11 mg of formulation, and then reweighed. The difference in weight was used as the amount of formulation applied. Commercial UV filters such as BP3, 4MBC, and AVO were used as reference standards. The filters were obtained from Sigma-Aldrich, Darmstadt, Germany.

The plate with tissues was embedded with parafilm and incubated at specific time points at 32 °C. As the passive diffusion of chemicals (and therefore their skin absorption) is affected by temperature, the diffusion chamber and skin were maintained at a constant temperature close to normal skin temperature of 32 ± 1 °C [19,23,30,32]. At fixed time points (0, 1, 2, 3, 4, 5, 6, and 24 h), aliquots of the receptor fluid (0.5 mL) were collected from the receptor compartment and immediately replaced with fresh fluid [19,30,32,67,68]. After 24 h the entire receptor fluid was collected, and the skin surface was washed thoroughly with cotton buds impregnated in methanol (5 times). The tissues were removed from the inserts and both the donor and receptor compartments were washed separately with methanol. The samples were stored at −20 °C until chemical analysis [30,32,67].

All samples were spiked with 25 µL of pentoxifylline (PTX, Sigma-Aldrich, Darmstadt, Germany) (final concentration of 20 µM) used as an internal standard prior to extraction [32]. The samples were analyzed using ultra high-pressure liquid chromatography coupled with mass spectrometry (UHPLC/MS; Waters Corporation, Milford, MA, USA). Quantification was based on peak area and calculated from standard calibration curves of the compound adjusted by the internal standard area. The assay was in agreement with the Organization for Economic Co-operation and Development (OECD) guideline 428 [19].

##### Method Validation

*Preparation of standards*

The analyzed compounds (JH3, JH10, BP3, 4MBC, and AVO) were weighed to 1 mg in a volumetric flask using analytical balance. The volume was brought to 1 mL using LC/MS-grade methanol to make 1000 μg/mL stock solutions. The solutions were stored at −20 °C and used to make dilutions for calibration curves. Pentoxifylline was weighed to 1 mg in a volumetric flask using analytical balance. The volume was brought to 1 mL using methanol to make a 1 mg/mL solution. The obtained solution was subsequentially diluted with methanol to obtain 400 μM internal standard solution. The solution was stored at −20 °C and used to make dilutions for calibration curves.

*Preparation of calibration samples*

The series of dilutions of the 1000 μg/mL solutions of the analyzed compounds were prepared by diluting 200 μL of the stock solutions of the investigated compounds with HPLC grade water to make 1 mL, and afterwards diluting 500 μL of the obtained solutions again with water to make 1 mL. The HPLC grade water was obtained from an HLP 5 (Hydrolab, Poland) apparatus and was filtered through a 0.2 µm filter before use. The procedure was repeated several times to obtain solutions with the concentration of the compounds in the range of 0.39–200 μg/mL. An amount of 50 μL of the pentoxifylline internal standard solution was added to the obtained solutions and diluted with water to make 1 mL. Finally, solutions with the concentration of the investigated compounds in the range of 0.20–100 μg/mL and pentoxifylline 20 μM were obtained.

*UPLC/UV-Vis-MS analysis*

The UPLC-MS/MS system consisted of Waters Acquity UPLC (Waters Corporation, Milford, MA, USA) coupled to Waters TQD mass spectrometer (electrospray ionization mode ESI-tandem quadrupole). Chromatographic separations were carried out using the Acquity UPLC BEH (bridged ethyl hybrid) C_18_ column; 2.1 × 100 mm, and 1.7 µm particle size, equipped with Acquity UPLC BEH C18 VanGuard pre-column; 2.1 × 5 mm, and 1.7 µm particle size. The column was maintained at 40 °C and eluted under gradient conditions using from 95% to 0% of eluent A over 10 min, at a flow rate of 0.3 mL/min. Eluent A: water/formic acid (0.1%, *v*/*v*); eluent B: acetonitrile/formic acid (0.1%, *v*/*v*). An amount of 10 µL of each sample was injected. LC/MS grade acetonitrile and formic acid were purchased from Sigma-Aldrich (Darmstadt, Germany). Chromatograms were recorded using Waters eλ PDA detector. Spectra were analyzed in the 200–700 nm range with 1.2 nm resolution and sampling rate 20 points/s. Data acquisition software was MassLynx (version 4.1, Waters).

The described method was validated for the determination of the analyzed compounds by UPLC/UV-Vis method according to ICH guidelines [69].

*Specificity*

To demonstrate the specificity of the developed UPLC/UV-Vis method, the solution containing all the investigated compounds was analyzed.

*System suitability*

Peaks of the analyzed compounds were well-resolved (R > 2) on all the observed chromatograms. The identity and purity of the obtained peaks were analyzed using the MS method.

*Linearity*

The linearity for the investigated compounds was assessed by injecting ten separately prepared solutions covering the range of 0.20–100 μg/mL of the investigated compounds. During the statistical analysis, the linear model and linearized nonlinear model (quadratic model) were analyzed:

Response = a_0_ + a_1_c (linear model), or

Response = a_0_ + a_1_c + a_2_c^2^ (quadratic model),

where Response = AUC/AUC_IS_, AUC—areas under the peaks of the investigated compounds on DAD chromatogram, AUC_IS_—area under the peak of pentoxifylline on DAD chromatogram.

The slope of the regression line, y-intercept, standard deviations of slope and intercept, correlation coefficient, r^2^ value, and standard error of residuals of the calibration curve were calculated using the program Statistica (version 13). Next, to determine whether the residuals have normal distribution, the Shapiro–Wilk statistical test was used. Additionally, the Mandel’s fitting test was performed to check the linearity of the calibration curve.

*Limit of detection (LOD) and limit of quantification (LOQ)*

Based on the standard error of residuals (Se), the parameters of the calibration plot and following the definition of limit of detection (LOD) and limit of quantification (LOQ), i.e., LOD–the concentration estimated for the response equal to 3.3 Se and LOQ–the concentration estimated for the response equal to 10 Se, the LOD and LOQ for examined compounds were estimated.

*Precision and accuracy*

The repeatability and accuracy of the method was checked by a six-fold analysis of the concentration level 50 μg/mL of the investigated compounds’ solutions. The same protocol was followed for three subsequent days to study the intermediate precision of the proposed method. The RSD (%) of the peaks areas were calculated. The statistical significance of interday differences was tested with ANOVA.

*Recovery*

Recovery of the method was assessed by analysis of the real sample spiked with the stock solutions of the compounds at level 20 μg/mL. The recovery was then calculated using the equation: [(c_spiked_–c_not spiked_)/20 µg/mL] × 100%, where c_spiked_ and c_not spiked_ are concentrations of the compounds in μg/mL obtained for the spiked and not spiked sample, respectively.

*Robustness*

To demonstrate the robustness of the method, deliberate small changes of flow rate, composition of eluents, and column temperature were made around the optimal values. The mobile phase flow rate was 0.30 mL/min; to study the effect of the flow rate on resolution, the flow rate was changed to 0.29 and 0.31 mL/min. The effect of the column temperature was studied at 36 and 44 °C (instead of 40 °C), and the mobile phase composition was changed +1% from the initial composition.

#### 4.3.2. The Episkin^TM^ Skin Irritation Test Method

Prior to the irritation experiments, the Episkin^TM^ inserts were washed with sterile PBS to remove traces of the MM and transferred into new 12-well plates containing 2 mL of fresh MM. In the irritation protocol, 5 µL of sterile water was applied onto the surface of the epidermis, followed by the application of 26 mg/cm^2^ of the formulations of test compounds (JH3 and JH10) using disposable flexible formulations. Sodium dodecyl sulphate (SDS; Sigma-Aldrich, Darmstadt, Germany) (5% in water) and PBS were used as positive and negative controls, respectively. Both controls were applied topically (26 mg/cm^2^). Samples were tested in two independent experiments using three tissues for each chemical, positive, or negative control.

The Episkin^TM^ model was incubated for 15 min at room temperature, and after the exposure time, was washed with PBS (25 mL). Then, the tissues were placed in new 12-well plates containing 2 mL of the MM per well and incubated for an additional 42 h at 37 °C in a humidified atmosphere of 5% CO_2_ as post-treatment incubation time. At the end of this period, the culture media of each tissue were collected and stored at −20 °C for Il-1α measurement.

The cell viability was assessed by a subsequent incubation of the tissues for 3 h with freshly prepared 3-(4,5-dimethylthiazol-2-yl)-2,5-diphenyltetrazolium bromide (MTT; Sigma-Aldrich, Darmstadt, Germany) solution (0.3 mg/mL in assay medium (AM)) in a 12-well plate. Then, a biopsy of the epidermis was done using a puncher (Episkin, Lyon, France), and the precipitated formazan was extracted using 500 µL of acidified isopropanol (Chempur, Piekary Śląskie, Poland) (0.04 N HCl in isopropanol) for 48 h (4 °C). At the end of the extraction period, 200 µL of each sample were transferred to 96-well plates for absorbance measurements at 570 nm (A570) [70,71,72,73]. The assay was in agreement with the OECD guideline 439 [16]. Viability (percent of control) was determined by dividing A570 of experimental wells by A570 of control wells × 100%. The test substance was considered as an irritant when mean tissue viability was equal or less than 50% [16,17].

Il-1α measurement was conducted by a classic enzyme-linked immunosorbent assay (ELISA) technique (Cayman Chemical, Ann Arbor, MI, USA). Firstly, 100 µL of the samples were added into a 96-well ready to use microtiter plate (precoated with a monoclonal antibody specific for Il-1α (Il-1α capture antibody). Secondly, 100 µL of an acetylcholinesterase:Fab’ Conjugate (AChE:Fab’) was added to each well. The plate was covered with plastic film and incubated overnight at 4 °C. At the end of the incubation period the contents of each well were removed and rinsed five times with wash buffer. Next, 200 µL of Ellman’s reagent (containing the substrate for AChE) was added to each well, the plate was covered with plastic film and developed in the dark using an orbital shaker. The absorbance of the wells was measured at 410 nm. Il-1α concentrations were then calculated using the standard curve.

#### 4.3.3. The Episkin^TM^ Phototoxicity Test Method

Following overnight incubation in MM at 37 °C in a humidified atmosphere of 5% CO_2_, the tissues were transferred to new 12-well plates containing 1.5 mL of PBS. Compounds JH3 and JH10 (final concentrations of 0.5 and 1 mM) solubilized in ethanol (final concentration of 1% in water) were applied topically on the tissue surface. Chlorpromazine (CPZ, chlorpromazine hydrochloride, C_17_H_19_ClN_2_S·HCl, CAS number: 69-09-0, molecular weight: 353.33, purity: meets USP testing specifications, Sigma-Aldrich, Darmstadt, Germany) (final concentration of 0.5 mM, based on literature data) was used as a positive control, whereas solvent alone served as a negative control.

Duplicate plates were prepared for UVA and dark (controls) exposure purposes. After the treatment period of 2 h, the excess test compound was removed from each well, and the tissues were exposed to UVA at a non-cytotoxic dose of 50 J/cm^2^ (6 h 23 min at 250 W/m^2^) with a solar light simulator equipped with a window glass filter and ID65 filter (Suntest CPS+, Atlas, Linsengericht, Germany). The emitted spectrum contained both UV and visible outputs with a UV cut-on of approx. 320 nm and corresponded to ID65 per ISO 10,977 [74]. The irradiance level between 320 and 400 nm was approximated by the following equation: W/m^2^ (320–400 nm) = W/m^2^ (300–800 nm) ÷ 11.5. In parallel, the non-irradiated treated tissues were placed in the dark under the same conditions. After a 2 h post-exposure period, both UV-exposed and non-exposed tissues were transferred to fresh MM and incubated for 22 h at 37 °C in a humidified atmosphere of 5% CO_2_ (post-treatment period) [17,75,76]. Next, cell viability was assessed using an MTT conversion test and the culture media aliquots were kept frozen (−20 °C) for Il-1α measurement (see 2.3.2. The Episkin^TM^ skin irritation test method section). Samples were tested in two independent experiments using three tissues for each chemical, positive, or negative control.

### 4.4. CHO-K1 Cytotoxicity Assay: Cell Viability Test

The Chinese hamster ovary CHO-K1 cell line was obtained from the American Type Culture Collection (ATCC CCL-61). The CHO-K1 cell line was cultured in a humidified incubator at 37 °C with 5% CO_2_, in F-12K Medium (Kaighn’s Modification of Ham’s F-12 Medium) (ATCC) supplemented with 10% fetal bovine serum (FBS; Gibco, Waltham, MA, USA) and antibiotics (1% streptomycin/penicillin salts mixture, Sigma–Aldrich, Darmstadt, Germany).

In the assay the maximum non-cytotoxic amounts of test compounds JH3 and JH10 and commercial UV filter 4MBC were determined. The cells were seeded at a density of 6 × 10^3^ on 96-well plates. Following overnight culture, the cells were then treated with increasing doses of test compounds (1–150 µM) for 24 h. Test compounds and 4MBC were dissolved in DMSO (solvent, final concentration 0.1%) and ethanol (co-solvent, final concentration 1%). At the end of the incubation period, 10 µL of MTT reagent was added to each well. After 4 h incubation (37 °C, 5% CO_2_), the medium was discarded, and the formazan produced in the cells appeared as dark crystals in the bottom of the wells. Next, pure DMSO was added to each well. The optical density data of converted dye was measured at 570 nm (A570) on microplate reader. Viability (percent of control) was determined by dividing A570 of experimental wells by A570 of control wells × 100% [15,77]. The experiment was conducted two times with three repetitions for each condition.

### 4.5. In Vitro Micronucleus Assay

The CHO-K1 cells were seeded in 6-well plates at a density of 1 × 10^6^ cells/well and incubated overnight at 37 °C, 5% CO_2_ atmosphere. Then, the incubation medium was replaced with fresh medium containing JH3, JH10, commercial UV filter 4MBC, or mitomycin C (MMC; Sigma Aldrich, Darmstadt, Germany) (0.5 µg/mL) as a positive control. The test compounds and 4MBC were dissolved in DMSO (solvent, final concentration 0.1%) and ethanol (co-solvent, final concentration 1%). After 24 h of treatment, the medium was removed, and the cells were rinsed with PBS. Then, fresh medium containing cytochalasin B (CytB; Sigma Aldrich, Darmstadt, Germany) (4.5 µg/mL) was added and incubation was continued for the next 24 h.

After the incubation period, the cells were washed with fresh medium, detached by trypsinization, and centrifuged. The supernatant was discarded and 5 mL of 1% ice-cold sodium citrate (Chempur, Piekary Śląskie, Poland) was added to the cell pellet, which was then gently resuspended. After 15 s, 5 mL of freshly prepared fixative solution (methanol/acetic acid, 3:1) and 4 drops of formaldehyde were added. The cell suspension was centrifuged (5 min, 1000 rpm), the supernatant was discarded again, and the pellet was fixed in fixative mixture two more times, without formaldehyde. After the third fixation, part of the supernatant was removed, and around 1 mL of the supernatant was retained to resuspend the cells. The suspension was then dripped onto clean, previously frozen, glass slides to dry. After air-drying, the slides were stained with Giemsa dye (1:20 in phosphate buffer) for 5 min [56,78,79].

One thousand binucleated cells per slide with intact nuclei of equal size, similar pattern of cytoplasm staining, intact membrane, and distinguishable from adjacent cells, without apoptotic and necrotic cells were analyzed. Only the micronuclei with the same morphology and color of the main nuclei, with 1/16 to 1/3 of the major nucleus diameter, unrefringent and not overlapping or connected to the main nuclei were counted. Moreover, the presence of dicentric bridges (DB) and nuclear buds (NB) in the binucleated cells was analyzed. The nuclear division index (NDI) was calculated using the following formula: [(1 × MOC) + (2 × BC) + (3 × MUC)]/*N*, where MOC is the number of mononuclear cells, BC is the number of binuclear cells, MUC represents the number of multinuclear cells, and *N* is the total number of scored cells. The micronucleus incidence was presented as a number of micronuclei per 1000 examined binuclear cells [78,79]. The experiment was conducted two times with three repetitions for each condition.

### 4.6. In Vitro Microsomal Biotransformation

The microsome incubation procedure was adapted from the previous studies [80,81]. Reaction mixtures comprising the test compound JH3 or JH10 (20 µM in methanol), human liver microsomes (HLMs; Sigma Aldrich, Darmstadt, Germany) (0.8 mg/mL), and 100 mM potassium phosphate buffer (pH 7.4) were preincubated before the addition of NADPH-regenerating system (NADP, glucose-6-phosphate and glucose-6-phosphate dehydrogenase in potassium phosphate buffer). The NADPH-regenerating system and buffer components were obtained from Sigma-Aldrich (Darmstadt, Germany). Incubation was conducted for different time points (i.e., 5, 15, and 30 min) with temperature set at 37 °C. The reaction was quenched with ice-cold methanol, followed by the addition of internal standard (IS)–PTX (20 µM). The mixture was then centrifuged.

Supernatant analysis was performed using UHPLC/MS (Waters Corporation, Milford, MA, USA). Two control samples were prepared with each incubation set. The first, a mixture of a test compound and potassium phosphate buffer, and without the addition of HLMs nor the NADPH-regenerating system, was used to exclude the possibility of hydrolysis facilitated by the buffer solution. The second, a mixture of a test compound, potassium phosphate buffer and HLMs, but without the NADPH-regenerating system solution, was designed to evaluate the production of metabolites in the presence of enzymes but the absence of NADPH [14,82]. The assays were repeated two times. The in vitro half time (t_1/2_) for JH3 was established from the slope of the linear regression of ln percent parent compound remaining against incubation time. Intrinsic clearance (Cl_int_) was obtained from the equation: Cl_int_ = (volume of incubation [µL] (i.e., 250 µL)/protein in the incubation [mg] (i.e., 0.2 mg)) × 0.693/t_1/2_ [83,84,85].

### 4.7. Data Analysis

All results were analyzed using the GraphPad Prism program (version 5.0), which was also utilized for the elaboration of figures and tables. Comparisons between the data were performed using the analysis of variance (ANOVA) test, followed by Tukey’s post hoc test when significant differences among treatments were found. The significance was set at *p* < 0.05 and the results were reported as means and standard deviations.

## 5. Conclusions

The results found in the present study showed that the compounds JH3 and JH10 might be candidates for further investigations directed at their use as potential UV filters, as they display no skin irritation and no phototoxicity. Both compounds did not markedly enhance the frequency of micronuclei in CHO-K1 cells in the micronucleus assay. Moreover, preliminary in vitro studies with liver microsomes demonstrated that hydrolysis appears to constitute their important metabolic pathway. As regards Episkin^TM^ permeability experiments, JH3 permeability was lower than or close to currently used UV filters, whereas JH10 had the potential to permeate the skin. Therefore, a restriction of this compound’s permeability should be obtained by choosing the right vehicle or by optimizing it, which definitely should be addressed in future studies.

## Figures and Tables

**Table 1:** Chemical structures and photoprotective activity of the title compounds obtained in 2% (*w*/*w*) macrogol formulations.

Comp.	Chemical Structure	SPF_in vitro_ ^a^	UVA PF	λ_c_	UVA/UVB Ratio
**JH3**	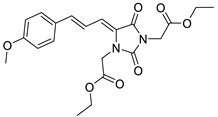	1.68 ± 0.04	6.83 ± 0.05	391	4.50 ± 0.32
**JH10**	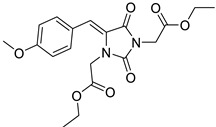	3.07 ± 0.04	2.59 ± 0.23	366	0.84 ± 0.08

^a^ SPF_in vitro_—sun protection factor.

**Table 2:** Regression analysis results—part 1.

Comp.	a_0_	a_1_	r	r^2^	LOD ^a^ (μg/mL)	LOQ ^b^ (μg/mL)
**BP3**	0.0082 ± 0.0032 (*p* < 0.01)	0.0512 ± 0.0023 (*p* < 10^−6^)	0.9992	0.9998	0.07	0.21
**4MBC**	0.0046 ± 0.0018 (*p* < 0.02)	0.0611 ± 0.0018 (*p* < 10^−6^)	0.9992	0.9998	0.05	0.15
**AVO**	0.0032 ± 0.0021 (*p* > 0.1)	0.0684 ± 0.0013 (*p* < 10^−6^)	0.9990	0.9998	0.10	0.30
**JH3**	0.0026 ± 0.0008 (*p* < 10^−2^)	0.0684 ± 0.0013 (*p* < 10^−6^)	0.9991	0.9998	0.03	0.09
**JH10**	−0.0011 ± 0.0002 (*p* < 10^−3^)	0.0421 ± 0.0003 (*p* < 10^−6^)	0.9998	0.9996	0.04	0.12

^a^ LOD—limit of detection, ^b^ LOQ—limit of quantification, BP3—benzophenone-3, 4MBC—4-methylbenzylidene camphor, AVO—avobenzone.

**Table 3:** Regression analysis results—part 2.

Comp.	Calibration Range (μg/mL)	Intraday RSD	Interday RSD	Accuracy	Recovery	Shapiro–Wilk Test for Residuals	Mandel’s Fitting Test
**BP3**	0.21–100	3.2%	4.1% (*p* > 0.6)	103.2%	93.8%	*p* > 0.5	*p* > 0.3
**4MBC**	0.15–100	3.9%	5.8% (*p* > 0.3)	101.0%	95.1%	*p* > 0.2	*p* > 0.4
**AVO**	0.30–100	2.1%	2.2% (*p* > 0.5)	98.9%	90.7%	*p* > 0.2	*p* > 0.2
**JH3**	0.09–100	2.2%	3.2% (*p* > 0.5)	102.4%	98.7%	*p* > 0.4	*p* > 0.3
**JH10**	0.12–100	1.4%	2.7% (*p* > 0.4)	100.7%	94.4%	*p* > 0.3	*p* > 0.1

BP3—benzophenone-3, 4MBC—4-methylbenzylidene camphor, AVO—avobenzone.

**Table 4:** Effect of JH3, JH10, and 4-methylbenzylidene camphor (4MBC) on the number of micronuclei (MN), dicentric bridges (DB), and nuclear buds (NB) on binucleated CHO-K1 cells and on the nuclear division index (NDI).

Treatments	Concentration (µM)	MN ^a^	DB ^a^	NB ^a^	NDI ^a^
**Normal control**	-	8.2 ± 3.1	5.5	4.1	1.45
**Vehicle control**	-	9.3 ± 4.3	4.6	5.2	1.41
**JH3**	1	10.1 ± 3.3	3.8	3.6	1.45
	5	10.5 ± 3.9	4.1	3.5	1.40
	10	12.0 ± 4.2	3.6	5.4	1.32
**JH10**	1	9.0 ± 3.9	3.5	4.3	1.31
	5	11.2 ± 3.6	3.8	5.2	1.38
	10	9.5 ± 3.7	3.5	3.2	1.47
**4MBC**	1	8.3 ± 2.9	4.6	3.7	1.35
	5	9.0 ± 3.4	4.5	3.5	1.30
	10	7.3 ± 4.4	3.4	4.9	1.39
**MMC**		88.5 ± 6.5 *	24.3 *	32.6 *	1.11 *

^a^ The aberrations count was performed in 1000 binucleated cells per slide. Vehicle control (DMSO 0.1%, ethanol 1%), positive control MMC (0.5 µg/mL). Results expressed as mean ± standard deviation. (*) *p* < 0.05 when compared to negative control (normal control and vehicle control). Experiment was conducted two times with three repetitions for each condition.

**Table 5:** Summary of data obtained for JH3 and JH10 and their ester hydrolysis products (M), generated in human liver microsomes (HLMs).

**Comp.**	**Parent** **Compound/Metabolite**	**ΔM**	**[M+H]^+^**	**Retention Time** **(min)**	**t_1/2_ ^a^** **(min)**	**Cl_int_ ^b^** **(µL/mg min)**	**Biotransformation**
**JH3**	Parent M	- −28	417 389	7.29 5.86	5 -	172 -	- Hydrolysis
**JH10**	Parent M	- −28	391 363	6.73 5.33	<<5 -	>>172 -	- Hydrolysis

^a^ t_1/2_—in vitro half time, ^b^ Cl_int_—intrinsic clearance.

## Data Availability

Not application.

## Sample Availability

Samples of the compounds JH3 and JH10 are available from the authors.

## References

[1] Maier T., Korting H.C. (2005). Sunscreens—Which and what for?. Skin Pharmacol. Physiol..

[2] Kim S., Choi K. (2014). Occurrences, toxicities, and ecological risks of benzophenone-3, a common component of organic sunscreen products: A mini-review. Environ. Int..

[3] Fabbrocini G., Triassi M., Mauriello M.C., Torre G., Annunziata M.C., de Vita V., Pastore F., D’Arco V., Monfrecola G. (2010). Epidemiology of skin cancer: Role of some environmental factors. Cancers.

[4] Durrer S., Maerkel K., Schlumpf M., Lichtensteiger W. (2005). Estrogen target gene regulation and coactivator expression in rat uterus after developmental exposure to the ultraviolet filter 4-methylbenzylidene camphor. Endocrinology.

[5] Schlumpf M., Kypke K., Vökt C.C., Birchler M., Durrer S., Faass O., Ehnes C., Fuetsch M., Gaille C., Henseler M. (2008). Endocrine active UV filters: Developmental toxicity and exposure through breast milk. Chimia.

[6] Gilbert E., Pirot F., Bertholle V., Roussel L., Falson F., Padois K. (2013). Commonly used UV filter toxicity on biological functions: Review of last decade studies. Int. J. Cosmet. Sci..

[7] Janjua N.R., Mogensen B., Andersson A.M., Petersen J.H., Henriksen M., Skakkebæk N.E., Wulf H.C. (2004). Systemic absorption of the sunscreens benzophenone-3, octyl-methoxycinnamate, and 3-(4-methyl-benzylidene) camphor after whole-body topical application and reproductive hormone levels in humans. J. Investig. Dermatol..

[8] Gonzalez H., Farbrot A., Larkö P., Wennberg A.M. (2006). Percutaneous absorption of the sunscreen benzophenone-3 after repeated whole-body applications, with and without ultraviolet irradiation. Br. J. Dermatol..

[9] Schauer U.M.D., Volkel W., Heusener A., Colnot T., Broshard T.H., von Landenberg F., Dekant W. (2006). Kinetics of 3-(4-methylbenzylidene)camphor in rats and humans after dermal application. Toxicol. Appl. Pharmacol..

[10] Giokas D.L., Salvador A., Chisvert A. (2007). UV filters: From sunscreens to human body and the environment. TrAC Trends Anal. Chem..

[11] Manová E., Von Goetz N., Hungerbühler K. (2014). Ultraviolet filter contact and photocontact allergy: Consumer exposure and risk assessment for octocrylene from personal care products and sunscreens. Br. J. Dermatol..

[12] Schneider S.L., Lim H.W. (2019). Review of environmental effects of oxybenzone and other sunscreen active ingredients. J. Am. Acad. Dermatol..

[13] Liang M., Yan S., Chen R., Hong X., Zha J. (2020). 3-(4-Methylbenzylidene) camphor induced reproduction toxicity and antiandrogenicity in Japanese medaka (*Oryzias latipes*). Chemosphere.

[14] Popiół J., Gunia-Krzyżak A., Piska K., Żelaszczyk D., Koczurkiewicz P., Słoczyńska K., Wójcik-Pszczoła K., Krupa A., Kryczyk-Poprawa A., Żesławska E. (2019). Discovery of novel UV-filters with favorable safety profiles in the 5-arylideneimidazolidine-2,4-dione derivatives group. Molecules.

[15] Popiół J., Piska K., Słoczyńska K., Bień A., Żelaszczyk D., Gunia-Krzyżak A., Koczurkiewicz P., Wójcik-Pszczoła K., Marona H., Pękala E. (2019). Microbial biotransformation of some novel hydantoin derivatives: Perspectives for bioremediation of potential sunscreen agents. Chemosphere.

[16] Organization for Economic Co-operation and Development (OECD) (2021). Organization for Economic Co-Operation and Development (OECD) Guideline No. 439: In Vitro Skin Irritation: Reconstructed Human Epidermis Test Method.

[17] Episkin. https://www.episkin.com.

[18] Organisation for Economic Co-operation and Development (OECD) (2011). Guidance Notes on Dermal Absorption.

[19] Organization for Economic Co-operation and Development (OECD) (2004). Organization for Economic Co-Operation and Development (OECD) Guideline No. 428: Skin Absorption: In Vitro Method.

[20] Netzlaff F., Kaca M., Bock U., Haltner-Ukomadu E., Meiers P., Lehr C.M., Schaefer U.F. (2007). Permeability of the reconstructed human epidermis model Episkin in comparison to various human skin preparations. Eur. J. Pharm. Biopharm..

[21] Monti D., Brini I., Tampucci S., Chetoni P., Burgalassi S., Paganuzzi D., Ghirardini A. (2008). Skin permeation and distribution of two sunscreens: A comparison between reconstituted human skin and hairless rat skin. Skin Pharmacol. Physiol..

[22] Samaras E.G., Riviere J.E., Ghafourian T. (2012). The effect of formulations and experimental conditions on in vitro human skin permeation-Data from updated EDETOX database. Int. J. Pharm..

[23] Abdallah M.A., Pawar G., Harrad S. (2015). Evaluation of 3D-human skin equivalents for assessment of human dermal absorption of some brominated flame retardants. Environ. Int..

[24] Klimová Z., Hojerová J., Beránková M. (2015). Skin absorption and human exposure estimation of three widely discussed UV filters in sunscreens-In vitro study mimicking real-life consumer habits. Food Chem. Toxicol..

[25] Tampucci S., Burgalassi S., Chetoni P., Monti D. (2018). Cutaneous permeation and penetration of sunscreens: Formulation strategies and in vitro methods. Cosmetics.

[26] Lehman P.A., Raney S.G. (2012). In vitro percutaneous absorption of ketoprofen and testosterone: Comparison of pluronic lecithin organogel vs. pentravan cream. Int. J. Pharm. Compd..

[27] Lenn J.D., Neil J., Donahue C., Demock K., Tibbetts C.V., Cote-Sierra J., Smith S.H., Rubenstein D., Therrien J.P., Pendergrast P.S. (2017). RNA aptamer delivery through intact human skin. J. Investig. Dermatol..

[28] Netzlaff F., Lehr C.M., Wertz P.W., Schaefer U.F. (2005). The human epidermis models EpiSkin, SkinEthic and EpiDerm: An evaluation of morphology and their suitability for testing phototoxicity, irritancy, corrosivity, and substance transport. Eur. J. Pharm. Biopharm..

[29] Schreiber S., Mahmoud A., Vuia A., Rübbelke M.K., Schmidt E., Schaller M., Kandárová H., Haberland A., Schäfer U.F., Bock U. (2005). Reconstructed epidermis versus human and animal skin in skin absorption studies. Toxicol. Vitro.

[30] Grégoire S., Patouillet C., Noé C., Fossa I., Benech Kieffer F., Ribaud C. (2008). Improvement of the experimental setup for skin absorption screening studies with reconstructed skin EPISKIN. Skin Pharmacol. Physiol..

[31] Rozman B., Gasperlin M., Tinois-Tessoneaud E., Pirot F., Falson F. (2009). Simultaneous absorption of vitamins C and E from topical microemulsions using reconstructed human epidermis as a skin model. Eur. J. Pharm. Biopharm..

[32] Abou-Elwafa Abdallah M., Pawar G., Harrad S. (2016). Human dermal absorption of chlorinated organophosphate flame retardants; implications for human exposure. Toxicol. Appl. Pharmacol..

[33] Schäfer-Korting M., Bock U., Gamer A., Haberland A., Haltner-Ukomadu E., Kaca M., Kamp H., Kietzmann M., Korting H.C., Krächter H.U. (2006). Reconstructed human epidermis for skin absorption testing: Results of the German prevalidation study. ATLA Altern. Lab. Anim..

[34] Durand L., Habran N., Henschel V., Amighi K. (2009). In vitro evaluation of the cutaneous penetration of sprayable sunscreen emulsions with high concentrations of UV filters. Int. J. Cosmet. Sci..

[35] Van Gele M., Geusens B., Brochez L., Speeckaert R., Lambert J. (2011). Three-dimensional skin models as tools for transdermal drug delivery: Challenges and limitations. Expert Opin. Drug Deliv..

[36] Pažoureková S., Hojerová J., Klimová Z., Lucová M. (2013). Dermal absorption and hydrolysis of methylparaben in different vehicles through intact and damaged skin: Using a pig-ear model in Vitro. Food Chem. Toxicol..

[37] Molins-Delgado D., Gago-Ferrero P., Díaz-Cruz M.S., Barceló D. (2016). Single and joint ecotoxicity data estimation of organic UV filters and nanomaterials toward selected aquatic organisms. Urban groundwater risk assessment. Environ. Res..

[38] PubChem. https://www.ncbi.nlm.nih.gov/pccompound.

[39] Liao C., Kannan K. (2014). Widespread occurrence of benzophenone-type UV light filters in personal care products from China and the United States: An assessment of human exposure. Environ. Sci. Technol..

[40] Watanabe Y., Kojima H., Takeuchi S., Uramaru N., Sanoh S., Sugihara K., Kitamura S., Ohta S. (2015). Metabolism of UV-filter benzophenone-3 by rat and human liver microsomes and its effect on endocrine-disrupting activity. Toxicol. Appl. Pharmacol..

[41] Scalia S., Mezzena M., Ramaccini D. (2011). Encapsulation of the UV filters ethylhexyl methoxycinnamate and butyl methoxydibenzoylmethane in lipid microparticles: Effect on in vivo human skin permeation. Skin Pharmacol. Physiol..

[42] Klimová Z., Hojerová J., Pažoureková S. (2013). Current problems in the use of organic UV filters to protect skin from excessive sun exposure. Acta Chim. Slovaca..

[43] Chatelain E., Gabard B., Surber C. (2003). Skin penetration and sun protection factor of five UV filters: Effect of the vehicle. Skin Pharmacol. Appl. Skin Physiol..

[44] Mestres J.P., Duracher L., Baux C., Vian L., Marti-Mestres G. (2010). Benzophenone-3 entrapped in solid lipid microspheres: Formulation and in vitro skin evaluation. Int. J. Pharm..

[45] Monti D., Tampucci S., Chetoni P., Burgalassi S., Saino V., Centini M., Staltari L., Anselmi C. (2011). Permeation and distribution of ferulic acid and its α-cyclodextrin complex from different formulations in hairless rat skin. AAPS Pharmscitech.

[46] Freitas J.V., Praça F.S., Bentley M.V., Gaspar L.R. (2015). Trans-resveratrol and beta-carotene from sunscreens penetrate viable skin layers and reduce cutaneous penetration of UV-filters. Int. J. Pharm..

[47] Monti D., Chetoni P., Burgalassi S., Tampucci S., Centini M., Anselmi C. (2015). 4-Methylbenzylidene camphor microspheres: Reconstituted epidermis (Skinethic^®^) permeation and distribution. Int. J. Cosmet. Sci..

[48] Mavon A., Raufast V., Redoulèst D. (2004). Skin absorption and metabolism of a new vitamin E prodrug, delta-tocopherol-glucoside: In vitro evaluation in human skin models. J. Control Release..

[49] Macfarlane M., Jones P., Goebel C., Dufour E., Rowland J., Araki D., Costabel-Farkas M., Hewitt N.J., Hibatallah J., Kirst A. (2009). A tiered approach to the use of alternatives to animal testing for the safety assessment of cosmetics: Skin irritation. Regul. Toxicol. Pharmacol..

[50] Kose O., Erkekoglu P., Sabuncuoglu S., Kocer-Gumusel B. (2018). Evaluation of skin irritation potentials of different cosmetic products in Turkish market by reconstructed human epidermis model. Regul. Toxicol. Pharmacol..

[51] Epstein J.H. (1983). Phototoxicity and photoallergy in man. J. Am. Acad. Dermatol..

[52] Kirsch-Volders M., Vanhauwaert A., Eichenlaub-Ritter U., Decordier I. (2003). Indirect mechanisms of genotoxicity. Toxicol. Lett..

[53] Chatterjee N., Walker G.C. (2017). Mechanisms of DNA damage, repair, and mutagenesis. Environ. Mol. Mutagen..

[54] Fenech M. (2000). The in vitro micronucleus technique. Mutat Res..

[55] Mateuca R., Lombaert N., Aka P.V., Decordier I., Kirsch-Volders M. (2006). Chromosomal changes: Induction, detection methods and applicability in human biomonitoring. Biochimie.

[56] Żelazna K., Rudnicka K., Tejs S. (2011). In vitro micronucleus test assessment of polycyclic aromatic hydrocarbons. Environ. Biotechnol..

[57] Scientific Committee on Consumer Products (SCCP) (2008). Opinion on 4-Methylbenzylidene Camphor (4-MBC).

[58] Chisvert A., León-González Z., Tarazona I., Salvador A., Giokas D. (2012). An overview of the analytical methods for the determination of organic ultraviolet filters in biological fluids and tissues. Anal. Chim. Acta.

[59] Guesmi A., Ohlund L., Sleno L. (2020). In vitro metabolism of sunscreen compounds by liquid chromatography-high resolution tandem mass spectrometry. Rapid Commun. Mass Spectrom..

[60] Lee S.K., Kim D.H., Yoo H.H. (2011). Comparative metabolism of sildenafil in liver microsomes of different species by using LC/MS-based multivariate analysis. J. Chromatogr. B Analyt. Technol. Biomed. Life Sci..

[61] Liederer B.M., Borchardt R.T. (2006). Enzymes involved in the bioconversion of ester-based prodrugs. J. Pharm. Sci..

[62] Laizure S.C., Herring V., Hu Z., Witbrodt K., Parker R.B. (2013). The role of human carboxylesterases in drug metabolism: Have we overlooked their importance?. Pharmacotherapy.

[63] Xie G., Zhou D.Y., Cheng K.W., Wong C.C., Rigas B. (2013). Comparative in vitro metabolism of phospho-tyrosol-indomethacin by mice, rats and humans. Biochem. Pharmacol..

[64] Tang J., Akao T., Nakamura N., Wang Z.T., Takagawa K., Sasahara M., Hattori M. (2007). In vitro metabolism of isoline, a pyrrolizidine alkaloid from Ligularia duciformis, by rodent liver microsomal esterase and enhanced hepatotoxicity by esterase inhibitors. Drug Metab. Dispos..

[65] Pękala E., Marona H. (2009). Estimating the lipophilicity of a number of 2-amino-1-cyclohexanol derivatives exhibiting anticonvulsant activity. Biomed. Chromatogr..

[66] Boyce C., Milborrow B.A. (1965). Simple assessment of partition data for correlating structure and biological activity using Thin-Layer Chromatography. Nature.

[67] Engesland A., Škalko-Basnet N., Flaten G.E. (2015). Phospholipid vesicle-based permeation assay and EpiSkin^®^ in assessment of drug therapies destined for skin administration. J. Pharm. Sci..

[68] Berthet A., Spring P., Vernez D., Plateel G., Hopf N.B. (2017). Ex vivo human skin permeation of methylchloroisothiazolinone (MCI) and methylisothiazolinone (MI). Arch. Toxicol..

[69] ICH-Q2 (R1) Validation and Analytical Procedures: Text and Methodology. Proceedings of the International Conference on Harmonization.

[70] Cotovio J., Grandidier M.H., Lelièvre D., Roguet R., Tinois-Tessonneaud E., Leclaire J. (2008). In vitro acute skin irritancy of chemicals using the validated EPISKIN model in a tiered strategy—Results and performances with 184 cosmetic ingredients. AATEX.

[71] Li N., Liu Y., Qiu J., Zhong L., Alépée N., Cotovio J., Cai Z. (2017). In vitro skin irritation assessment becomes a reality in China using a reconstructed human epidermis test method. Toxicol. Vitro.

[72] Pedrosa T.D.N., Catarino C.M., Pennacchi P.C., Assis S.R., Gimenes F., Consolaro M.E.L., Barros S.B.M., Maria-Engler S.S. (2017). A new reconstructed human epidermis for in vitro skin irritation testing. Toxicol. Vitro.

[73] Alépée N., Grandidier M.H., Cotovio J. (2019). Usefulness of the EpiSkin™ reconstructed human epidermis model within Integrated Approaches on Testing and Assessment (IATA) for skin corrosion and irritation. Toxicol. Vitro.

[74] (1993). Photography—Processed Photographic Colour Films and Paper Prints—Methods for Measuring Image Stability.

[75] Portes P., Pygmalion M.J., Popovic E., Cottin M., Mariani M. (2002). Use of human reconstituted epidermis Episkin for assessment of weak phototoxic potential of chemical compounds. Photodermatol. Photoimmunol. Photomed..

[76] Lelièvre D., Justine P., Christiaens F., Bonaventure N., Coutet J., Marrot L., Cotovio J. (2007). The EpiSkin phototoxicity assay (EPA): Development of an in vitro tiered strategy using 17 reference chemicals to predict phototoxic potency. Toxicol. Vitro.

[77] Słoczyńska K., Koczurkiewicz P., Piska K., Powroźnik B., Wójcik-Pszczoła K., Klaś K., Wyszkowska-Kolatko M., Pękala E. (2019). Similar safety profile of the enantiomeric N-aminoalkyl derivatives of *trans*-2-aminocyclohexan-1-ol demonstrating anticonvulsant activity. Molecules.

[78] Oliveira M.C., Lemos L.M., de Oliveira R.G., Dall’Oglio E.L., de Sousa Júnior P.T., de Oliveira Martins D.T. (2014). Evaluation of toxicity of Calophyllum brasiliense stem bark extract by in vivo and in vitro assays. J. Ethnopharmacol..

[79] Pavan E., Damazo A.S., Lemos L.M.S., Adzu B., Balogun S.O., Arunachalam K., Martins D.T.O. (2018). Evaluation of genotoxicity and subchronic toxicity of the standardized leaves infusion extract of Copaifera malmei Harms in experimental models. J. Ethnopharmacol..

[80] Słoczyńska K., Pańczyk K., Waszkielewicz A.M., Marona H., Pękala E. (2016). In vitro mutagenic, antimutagenic, and antioxidant activities evaluation and biotransformation of some bioactive 4-substituted 1-(2-methoxyphenyl)piperazine derivatives. J. Biochem. Mol. Toxicol..

[81] Słoczyńska K., Wójcik-Pszczoła K., Canale V., Żmudzki P., Zajdel P., Pękala E. (2018). Biotransformation of 4-fluoro-N-(1-{2-[(propan-2-yl)phenoxy]ethyl}-8-azabicyclo[3.2.1]octan-3-yl)-benzenesulfonamide, a novel potent 5-HT_7_ receptor antagonist with antidepressant-like and anxiolytic properties: In vitro and in silico approach. J. Biochem. Mol. Toxicol..

[82] Presley B.C., Castaneto M.S., Logan B.K., Jansen-Varnum S.A. (2020). Assessment of synthetic cannabinoid FUB-AMB and its ester hydrolysis metabolite in human liver microsomes and human blood samples using UHPLC-MS/MS. Biomed. Chromatogr..

[83] Singh J.K., Solanki A., Shirsath V.S. (2012). Comparative in-vitro intrinsic clearance of imipramine in multiple species liver microsomes: Human, rat, mouse and dog. J. Drug Metab. Toxicol..

[84] Leth-Petersen S., Bundgaard C., Hansen M., Carnerup M.A., Kehler J., Kristensen J.L. (2014). Correlating the metabolic stability of psychedelic 5-HT2A agonists with anecdotal reports of human oral bioavailability. Neurochem. Res..

[85] Słoczyńska K., Gunia-Krzyżak A., Koczurkiewicz P., Wójcik-Pszczoła K., Żelaszczyk D., Popiół J., Pękala E. (2019). Metabolic stability and its role in the discovery of new chemical entities. Acta Pharm..

